# Proving of Alumina

**Published:** 1889-08

**Authors:** E. W. Berridge

**Affiliations:** London


### PROVINGS OF ALUMINA.

E. W. Berridge, M. D., London.

*First proving.*—Miss C. G. took several doses of *Alumina*$^{cm}$ (Fincke). For five years she had had a pain in stomach-pit like a gnawing toothache, worse after food; it came on every summer; worst in June, July, and August, after which it gradually ceased. She had been treated by a mongrel, who declared she was incurable. The *Alumina* cured her after a severe aggravation.

It caused the following symptoms: Urine more frequent, copious, darker. At intervals sudden sharp pains like a stab in lower part of back, making her feel for the moment as if she could not straighten her spine. Low spirits; very trifling things presented themselves in a black light and seemed insurmountable. Upper lip covered with little blisters. Darting pains every now and then, first through one shoulder then in the other. A darting pain occasionally nearly at bottom of back on left side, which, while it lasts, stops the breath. Occasional dim sight.

*Second proving.*—At half-past twelve o'clock P. M., I took ten globules of CM (Fincke). Next day shooting in right side of neck rather posteriorly.

*Third proving.*—Mr. J. F. B. took one dose of *Alumina*$^{cm}$ (Fincke), for some chronic symptoms, August 22d, 1871, in morning. Next day had the following group of symptoms which were quite new to him:

In morning, when walking in room, feeling of faintness, extreme nausea, going off after breakfast. All day easily tired, inclined to lie down, yawning and stretching, drowsy, dullness of thought, flushes of heat, lassitude after talking, for which he had no inclination. Evening after sunset, fullness of head relieved by lying down.

